# MicroRNA changes in maternal serum from pregnancies complicated by twin‐twin transfusion syndrome: A discovery study

**DOI:** 10.1002/pd.5475

**Published:** 2019-06-18

**Authors:** Fiona L. Mackie, Bernadette C. Baker, Andrew D. Beggs, Agata Stodolna, Rachel Katie Morris, Mark D. Kilby

**Affiliations:** ^1^ Birmingham Women's and Children's NHS Foundation Trust and Institute of Metabolism and Systems Research University of Birmingham Birmingham UK; ^2^ Maternal and Fetal Health Research Centre University of Manchester Manchester UK; ^3^ Institute of Cancer and Genomic Science University of Birmingham Birmingham UK; ^4^ Fetal Medicine Centre, Birmingham Women's and Children's NHS Foundation Trust and Institute of Metabolism and Systems Research University of Birmingham Birmingham UK

## Abstract

**Objective:**

MicroRNAs (miRNAs) are used as biomarkers in cardiovascular disease and cancer. miRNAs are involved in placental development but have not previously been investigated in twin‐twin transfusion syndrome (TTTS). Our aim is to explore the miRNA profile of TTTS pregnancies.

**Method:**

Initial miRNA profiling was performed using a reverse transcription polymerase chain reaction (RT‐PCR) panel on maternal serum samples taken from five women prior to fetoscopic laser ablation for TTTS and compared with serum samples from five women with uncomplicated monochorionic diamniotic twin pregnancies. Validation RT‐PCR was performed in an additional cohort of eight TTTS pregnancies and eight uncomplicated pregnancies.

**Results:**

Median gestational age at sampling in the TTTS and control groups was 20^+0^ weeks (interquartile range [IQR], 19^+4^‐20^+0^) and 20^+2^ weeks (IQR, 20^+0^‐20^+2^), respectively. All samples passed quality control. One control sample was excluded as a biological outlier. Thirty‐one of 752 miRNAs were significantly different: 17 were upregulated and 14 downregulated in the TTTS group, although they did not remain significant following Benjamini‐Hochberg correction for multiple testing. The six miRNAs chosen for validation demonstrated no significant difference.

**Conclusion:**

This is the first study to investigate miRNA changes in TTTS pregnancies. We did not demonstrate a statistically significant difference in miRNAs in TTTS pregnancies, but further investigation is required.

**What's already known about this topic?**

- Twin‐twin transfusion syndrome (TTTS) is a common complication of monochorionic twins associated with high perinatal mortality and morbidity if untreated.
- TTTS has its origins in the monochorionic placenta with “conjoining” of the twin fetal circulations by anastomoses.
- Screening for TTTS relies upon regular, sequential ultrasound examination of the pregnancy observing for amniotic fluid discordance.
- MicroRNAs have been implicated in pregnancy‐related placental disease and may act as potential biomarkers in pregnancy complications.

**What does this study add?**

- This is the first study to explore microRNAs in TTTS. In maternal serum, the six microRNAs that were validated did not demonstrate a statistically significant difference in pregnancies complicated by TTTS, compared with uncomplicated monochorionic diamniotic twin pregnancies.
- Further investigation into the potential role of microRNAs in TTTS is warranted, particularly in different tissue samples.

## 1 INTRODUCTION

Twin‐twin transfusion syndrome (TTTS) complicates up to 15% of monochorionic diamniotic (MCDA) twins and if untreated is associated with a perinatal mortality of 80% to 90%^1^ and significant neurodevelopmental morbidity.^2^ The “donor” is hypoperfused and hypotensive with associated poor urine output and oligohydramnios, while the “recipient” has a hyperdynamic circulation, polyuria, and polyhydramnios with a high prevalence of cardiac dysfunction.^3^, ^4^ TTTS is associated with chronic unidirectional intertwin blood flow through monochorionic placental arteriovenous anastomoses (AVAs) leading to the hemodynamic imbalance within the twin fetal circulations.^5^

Presently, screening for TTTS involves the use of serial ultrasound, primarily to detect an imbalance of amniotic fluid in the monochorionic twins.^6^ Such ultrasound screening is workforce intensive, and a high proportion of MCDA twin pregnancies will not develop TTTS or growth restriction but will still undergo ultrasound screening every 2 weeks throughout pregnancy. However, timely detection of TTTS allows treatment by fetoscopic laser ablation (FLA) of the placental vascular anastomoses with a potential to modify the underlying disease process and significantly improve outcome.^7^ The theoretical use of maternal circulating biomarkers in screening for TTTS in its preclinical state and aiding in the definition of adverse pregnancy outcome in a monochorionic twin pregnancy would be beneficial, either alone or in combination with ultrasound findings.^8^, ^9^

MicroRNAs (miRNAs) are highly ubiquitous noncoding RNAs, consisting of a single strand of around 21 to 25 nucleotides that are conserved across many species.^10^ They are involved in posttranscriptional regulation of gene expression and function through base‐pairing with the 3′ untranslated region (UTR) of the complementary messenger RNA (mRNA) molecules, which then silences the mRNA by degradation of the target mRNA, or repression of translation.^11^, ^12^ These endogenous regulatory miRNAs are present in many human bodily fluids and tissues, including the human placenta.^13^, ^14^, ^15^, ^16^, ^17^, ^18^, ^19^ Their potential role as biomarkers in detecting and defining prognosis in heart disease and cancer has been reported,^20^ although there is less miRNA research in pregnancy, particularly twin pregnancy. In pregnancy, miRNAs appear important in human placentation affecting trophoblast proliferation, angiogenesis, and fetal growth.^21^, ^22^ Placental miRNA is known to be present, and stable, in maternal plasma^23^ and is believed to be released from the syncytiotrophoblast and carried in the plasma in at least two forms: protein‐bound miRNA and vesicular miRNA.^24^ They are cleared from the maternal circulation soon after delivery.^25^ Previous studies have demonstrated miRNA changes in maternal plasma in preeclampsia,^26^, ^27^, ^28^, ^29^, ^30^, ^31^, ^32^, ^33^, ^34^ in miRNAs thought to be related to angiogenesis. Indeed, certain miRNAs (miR‐195‐5p) may be linked to the increase in sFLT‐1 seen in preeclamptic pregnancies.^35^ Thus, miRNAs may act as “biomarkers” of placental and vascular function in pregnancy. There have been no previous reports of miRNAs in TTTS pregnancies. We hypothesized that there was a difference in human maternal serum miRNAs in pregnancies complicated with TTTS compared with uncomplicated matched MCDA twin pregnancies and that there is the potential for these molecules to be used as “biomarkers” of disease.

## 2 METHODS

### 2.1 Patients

Patients were recruited prospectively as part of the Optimal Management of Monochorionic Twins (OMMIT) study into an “investigation” or “validation” cohort.^8^ Briefly, maternal blood samples were obtained the day prior to FLA from patients attending the West Midlands Fetal Medicine Centre for FLA for the treatment of TTTS from August 2015 to August 2017. TTTS was defined as a maximum pool depth (MPD) > 8 cm in the recipient twin at less than 20 weeks gestation, or MPD >10 cm at greater than 20 weeks gestation, in combination with a MPD < 2 cm in the donor twin. Patients were prospectively staged using the Quintero classification.^36^ A control group of pregnant women with uncomplicated MCDA twin pregnancies who booked at Birmingham Women's and Children's NHS Foundation Trust were recruited over the same time period for the investigation work and validation work. Women were only included once chorionicity was confirmed by the presence of the “T” sign on first trimester ultrasound.^37^ Women whose pregnancies were affected by chromosomal/structural anomalies were not eligible for inclusion. Participants in the control group had no serious adverse maternal or fetal outcome and delivered two healthy babies, which did not require neonatal unit admission. Patients were matched based on maternal age, ethnicity, parity, body mass index (BMI), gestation at blood sampling, and fetal sex. This study received ethical approval from East Midlands Research Ethics Committee (15/EM/0244) in 2015, and all patients provided written informed consent.

### 2.2 Blood samples

Venous blood samples were collected in 7.5‐mL serum gel tubes (Sarstedt, Nümbrecht, Germany) from the antecubital fossa, allowed to clot for 1 hour at room temperature, centrifuged at 3000 *g* for 10 minutes at room temperature, and sediment‐free serum aliquots were stored immediately at −80°C prior to analysis and during transit. Serum sample aliquots were stored for a maximum of 13 months prior to analysis, which is considered acceptable practice in a recent best practice paper.^38^

### 2.3 Investigation profiling array

Samples taken from women in the investigation cohort were used to perform the initial investigation profiling array.

### 2.4 RNA extraction

Total RNA was extracted from serum using the miRCURY RNA Isolation Kit—Biofluids (Exiqon, Vedbaek, Denmark) as per the manufacturer's instructions (See Appendix A). The extracted RNA was stored at −80°C prior to transfer to Exiqon, Denmark, 2 months later, on dry ice.

### 2.5 Quality control assessment of samples

Hemolysis was assessed by two miRNAs known to correlate with the degree of hemolysis: hsa‐miRNA‐451, which is expressed on red blood cells, and hsa‐miRNA‐23a, which is not affected by hemolysis. A ratio > 7.0 of those two miRNAs indicates that the sample may be affected by hemolysis. Spike‐in (RNA isolation control UniSp2, UniSp4, UniSp5, and cDNA synthesis control UniSp6) was added to each sample as technical controls to assess extraction, reverse transcription, and qPCR. A negative control, “no template” sample, was included in the reverse transcription step to detect RNA contamination.

### 2.6 Quantitative real‐time polymerase chain reaction for miRNAs

miRNA profiling was performed by Exiqon using the miRCURY LNA Universal RT microRNA PCR Human panel I + II. These panels contain primer sets for the 752 human miRNA most commonly differentially expressed in disease and/or cited in the scientific literature, including miRNAs associated with cardiovascular disease, renal disease, angiogenesis, and placenta‐specific miRNA, in‐keeping with the pathophysiological mechanisms of TTTS (see Appendix B for full list of miRNAs tested). All the miRNAs were polyadenylated and reverse transcribed into cDNA. The cDNA and ExiLENT SYBR Green master mix were transferred to the qPCR panels with 384‐well plates preloaded with the specified primers, using a pipetting robot. The amplification was performed in a Lightcycler 480 Instrument (Roche).

### 2.7 Validation with RT‐PCR of candidate miRNAs

From the initial profiling array, eight candidate miRNAs were identified for validation based on being significantly different in the TTTS compared with control samples, prior to Benjamini‐Hochberg correction for multiple testing; being present in all investigation cohort samples; having validated targeted functional genes with strong evidence of miRNA‐target interaction (MTI) reported on MiRTarBase (version 7.0)^39^; and biological plausibility for association with TTTS based on a literature search using keywords: placenta, pregnancy, twin, maternal, gestation, cardio‐$, renal‐$, vascul‐$, angio‐$, trophoblast, and fetal.

miRNA was extracted from serum with miRNeasy Serum/Plasma Kit (Qiagen, Manchester, UK) according to manufacturer's instructions. The quality and the concentration of the samples were investigated with High Sensitivity RNA TapeStation (Agilent, Stockport, UK). Two endogenous controls were used: miR‐361‐5p and miR‐451a.

Samples were converted to cDNA, and miRNA was amplified using TaqMan Advanced miRNA cDNA Synthesis Kit (Applied Biosystems, Thermo Fisher Scientific, Warrington, UK) according to the manufacturer's instructions. Reverse transcription polymerase chain reaction (RT‐PCR) was performed with TaqMan Advanced miRNA Assays (Applied Biosystems) according to the manufacturer's instructions. Five microliters of 1:10 dilution of cDNA template was used for the RT‐PCR reaction with 10 μL of 2× TaqMan Fast Advanced Master Mix (Applied Biosystems), 1 μL of 20× TaqMan Advanced miRNA Assay, and 4 μL of RNase‐free water. The reaction was run on the QuantStudio 5 instrument (Applied Biosystems) under conditions: one cycle for 20 seconds at 95°C and then 40 cycles of 1 second at 95°C and 20 seconds at 60°C.

### 2.8 Statistical analysis

Normalization was performed based on the average of the assays detected in all 10 samples. A heatmap with red denoting miRNA expression above the mean and green denoting miRNA expression below the mean, and a principal component analysis (PCA) plot were drawn to assess the miRNA expression profiles of the samples by depicting the normalized dCq values for the top 50 miRNAs with the widest variation in expression in the samples. If any samples appeared to be outliers, this was further investigated as to whether the cause was due to pathology, sample quality, an error in processing, or natural miRNA variation. To compare the miRNAs in different groups, a volcano plot was drawn to allow easy identification of miRNAs upregulated and downregulated in each group. To quantitatively compare the groups, normality of the data was assessed using the Shapiro‐Wilk test. The *t* test was used when data were parametric, and the Wilcoxon test or Mann‐Whitney *U* test was used when data were nonparametric to compare matched samples, or groups accordingly. If any significant differences were seen, the validated miRNA target interactions were investigated of the five most significantly upregulated and five most significantly downregulated miRNAs, using miRTarBase (version 7.0).^39^ Benjamini‐Hochberg correction was applied to control for the false discovery rate in the context of multiple testing. *P* < .05 was considered significant. In order to further investigate the differential expression differences and correct for population substructure, raw dCt data on a probe‐wise level was imported into R 3.3.1 (R Core Team (2013). R: A language and environment for statistical computing (R Foundation for Statistical Computing, Vienna, Austria)), and multivariate logistic regression analysis was performed with case control as the outcome and age, ethnicity, gestation, parity, and maternal BMI as independent variables along with dCt using *limma*. Moderated ‐statistics were then calculated using empirical Bayesian shrinkage (*eBayes)* and the *topTable* command used to rank significant probes by descending Bayes factor (BF).To assess the validation cohort, individual data points were plotted on Stata using the *dotplot* command.^40^ The ΔΔCts of the control group were compared with the matched ΔΔCts of the TTTS group by the Wilcoxon signed rank test on using the *signrank* command.^41^ Findings with *P* < .05 were considered significant.

For the initial profiling array, no power calculation was performed as it was discovery work, thus a sample size of 10 is considered adequate, based on existing literature. The results of the investigation cohort were used to perform a power calculation based on the observed dCt values, standard deviations and correcting for multiple testing (using Stata 12.1) and revealed that 16 samples (n = 8 TTTS, n = 8 control) are needed in the validation cohort to demonstrate a statistically significant difference between the TTTS and uncomplicated MC twin pregnancy samples, with close matching of demographic variables in subjects, as in the investigation cohort.

## 3 RESULTS

### 3.1 Monochorionic twin pregnancy demographic data

#### 3.1.1 Investigation cohort

In the investigation cohort, five patients were included in the TTTS group and were matched to five from the control group: 2001 was paired with 2047, 2002 with 2020, 2018 with 2027, 2022 with 2039, 2043 and 2023. TTTS patients were selected pragmatically to represent different outcomes of TTTS and matched as closely with the control group as possible. There was no significant difference in the patient demographics of the two groups (Table 1). The median gestational age at blood sampling in the TTTS group was 20^+0^ weeks (IQR, 19^+4^‐20^+0^ weeks) compared with 20^+2^ weeks (20‐20^+2^) in the control group.

**Table 1:** Investigation cohort demographic data

Patient Number	Complication (Quintero Stage)	Maternal Age, y	Maternal Ethnicity	Parity	Maternal BMI, kg/m^2^	GA at Blood Sampling, wk	Fetal Sex
2001	Nil	24	White European	0	31.9	19^+1^	Female
2047	TTTS (Stage 2)	29	White European	1	29.4	20^+0^	Female
2002	Nil	27	White European	1	25.2	19^+4^	Female
2020	TTTS (Stage 3R)	33	White European	1	15.8	19^+5^	Female
2018	Nil	24	White European	0	24.7	20^+0^	Female
2027	TTTS (Stage 3R)	23	White European	1	32	20^+3^	Male
2022^a^	Nil	28	White European	0	29.4	20^+0^	Female
2039	TTTS (Stage 2)	28	White European	0	19.6	20^+2^	Female
2043	Nil	29	White European	0	18.5	21^+1^	Male
2023	TTTS (Stage 2)	35	White European	0	25.1	20^+2^	Male

Abbreviations: BMI, body mass index; GA, gestational age; TTTS, twin‐twin transfusion syndrome.

a This patient was excluded from analysis as a biological outlier due to natural variation in maternal miRNA.

#### 3.1.2 Validation cohort

An independent cohort of 19 women in total was included in the validation cohort. The sample from participant 2124 was used as the reference sample as it most closely matched the median characteristics of the validation control group. Samples 2102 and 2013 (TTTS samples) were removed from analysis as there was insufficient miRNA in the samples; thus, there were eight control samples and eight TTTS samples (Table 2); 2015 was paired with 2067, 2045 with 2025, 2068 with 2071, 2094 with 2069, 2104 with 2012, 2108 with 2101, 2112 with 2009, 2086 and 2017. TTTS patients were selected to match the control group as closely as possible. The median gestational age at blood sampling in the TTTS group was 20^+1^ weeks (IQR, 19^+6^‐20^+5^ wk) compared with 20^+2^ weeks (20^+0^‐20^+6^ wk) in the control group.

**Table 2:** Validation cohort demographic data

Patient Number	Complication (Quintero Stage)	Maternal Age, y	Maternal Ethnicity	Parity	Maternal BMI, kg/m^2^	GA at Blood Sampling, wk	Fetal Sex
2015	Nil	42	White European	4	44.5	20^+0^	Female
2067	TTTS (Stage 3)	27	White European	1	35.7	19^+4^	Female
2045	Nil	20	White European	2	19.0	21^+5^	Male
2025	TTTS (Stage 2)	22	White European	2	18.3	20^+4^	Male
2068	Nil	36	White European	1	25.5	19^+6^	Female
2071	TTTS (Stage 1)	35	White European	1	22.7	20^+0^	Female
2094	Nil	30	White European	0	27.1	20^+3^	Female
2069	TTTS (Stage 3)	28	White European	1	23.5	20^+1^	Female
2104	Nil	36	White European	1	25.6	20^+4^	Male
2012	TTTS (Stage 3)	27	White European	3	23.5	20^+1^	Male
2108	Nil	34	White European	0	29.1	19^+2^	Male
2101	TTTS (Stage 2)	24	White European	1	23.9	21^+0^	Female
2112	Nil	36	White European	1	24.6	20^+0^	Male
2009	TTTS (Stage 2)	24	White European	1	27.6	19^+4^	Male
2086	Nil						
2017	TTTS (Stage 3)	28	White European	4	29.8	21^+3^	Female
2124 (reference)	Nil	28	White European	1	31.7	20^+2^	Female
2102^a^	TTTS (Stage 3)	21	White European	0	28.6	19^+1^	Male
2013^a^	TTTS (Stage 3)	20	White European	1	19.8	19^+5^	Female

Abbreviations: BMI, body mass index; GA, gestational age; TTTS, twin‐twin transfusion syndrome.

a Patient excluded from analysis as insufficient miRNA.

#### 3.1.3 Quality control of investigation profiling array samples

**Figure 1:**
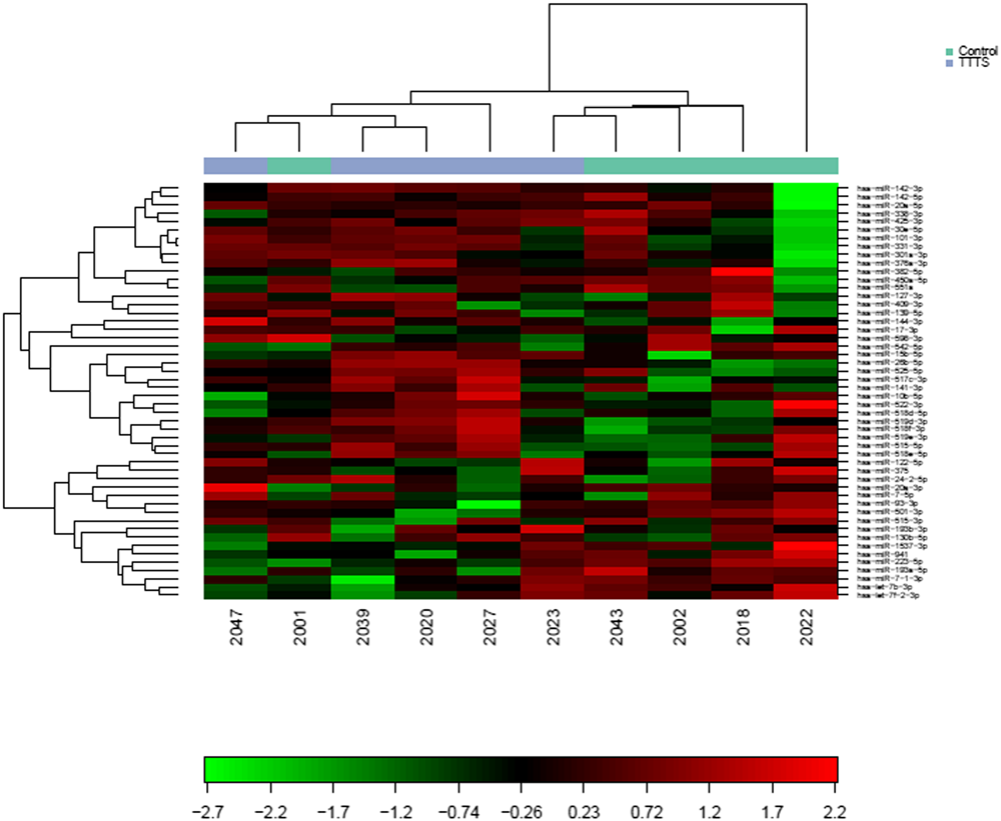
Heatmap of miRNA expression in all maternal serum samples from pregnancies with twin‐twin transfusion syndrome (TTTS) compared with matched controls with uncomplicated monochorionic diamniotic twin pregnancies [Colour figure can be viewed at wileyonlinelibrary.com]

**Figure 2:**
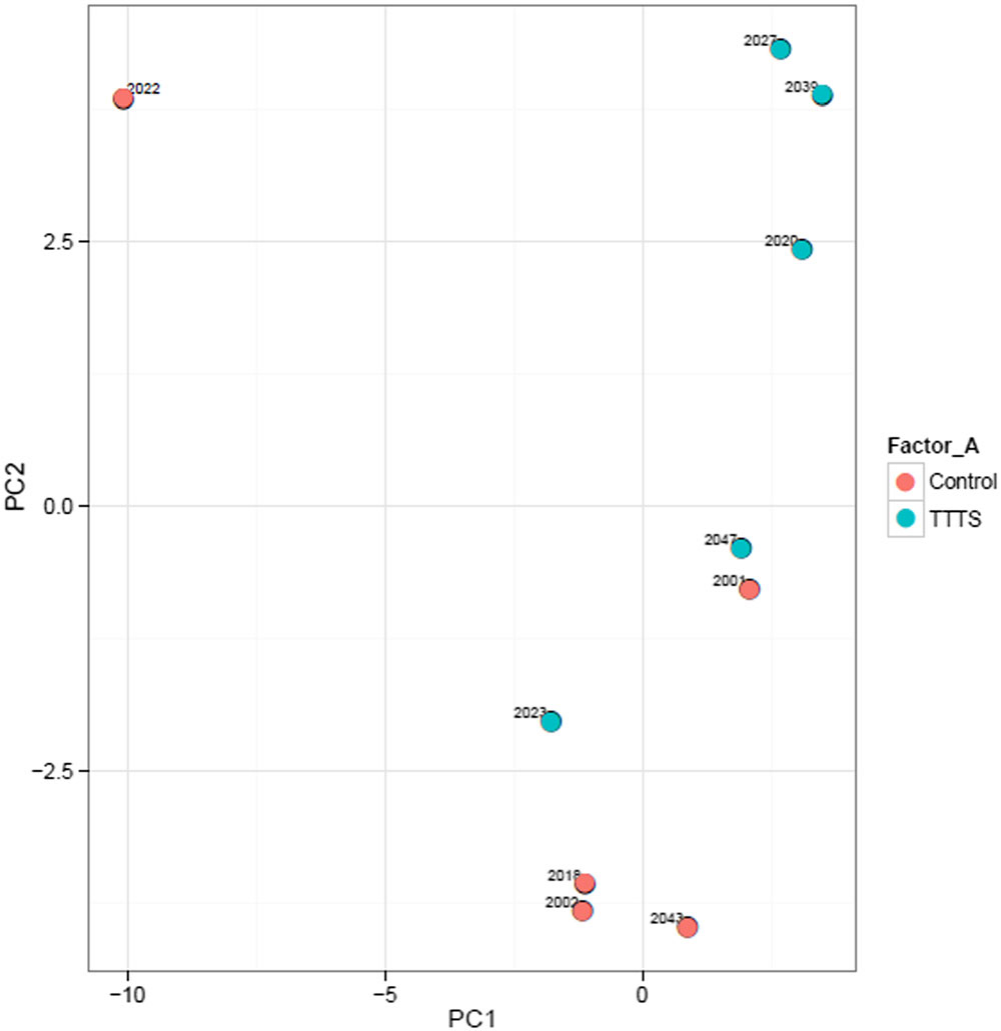
Principal component analysis (PCA) plot of top 50 miRNA with largest variation in expression across all maternal serum samples from pregnancies with twin‐twin transfusion syndrome (TTTS) compared with matched controls with uncomplicated monochorionic diamniotic twin pregnancies [Colour figure can be viewed at wileyonlinelibrary.com]

There was a low level of hemolysis in all 10 samples, and the steady level of the spike‐ins in the samples demonstrated high‐quality input RNA and successful reverse transcription and qPCR (data available on request). From the heatmap and PCA plot of the top 50 miRNA with largest variation in expression across all samples (Figures 1 and 2), sample 2022 (control) appeared to be an outlier. When this was further investigated, the quality of the sample was the same as the other samples, and there was no clinical reason why this sample should be different; therefore, the difference was believed to be due to biological variation, and the sample was excluded from further analysis.

#### 3.1.4 Detection of differential miRNAs in TTTS and control patients in profiling array samples

**Figure 3:**
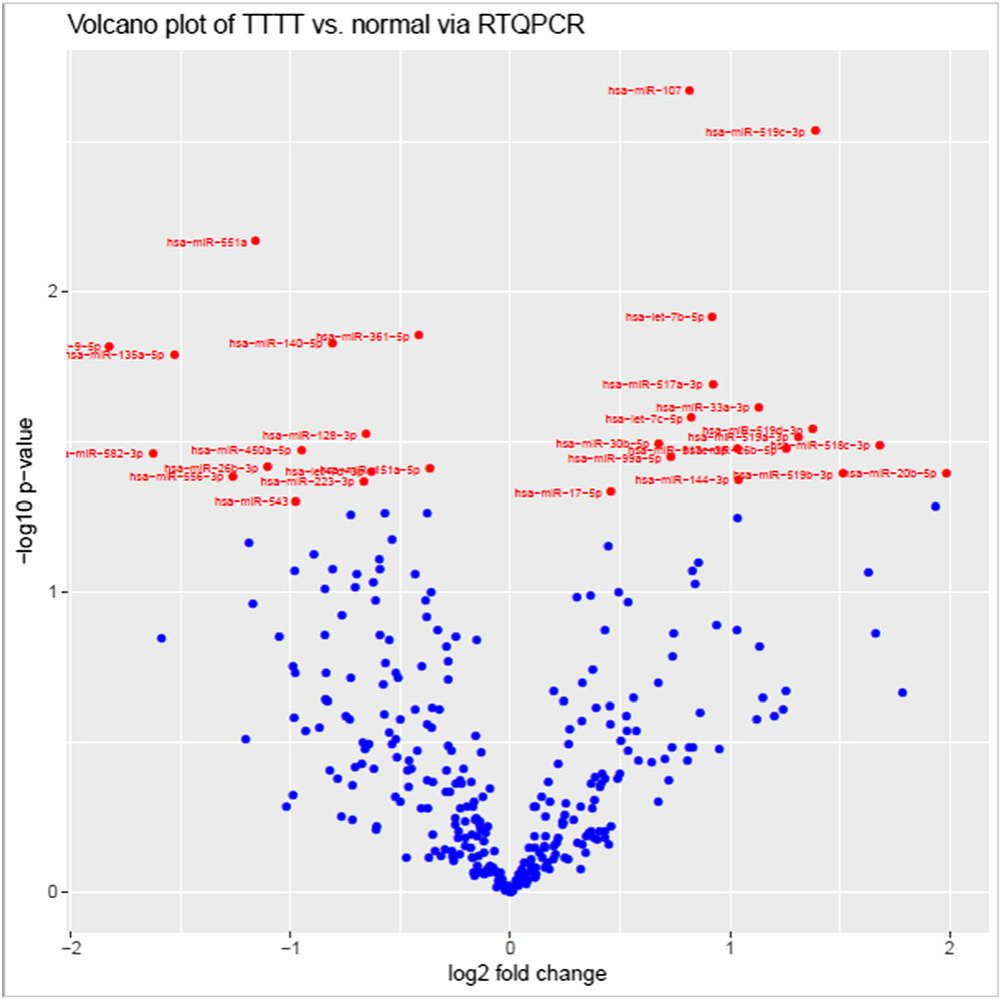
Volcano plot comparing fold change of miRNA expression in maternal serum samples from pregnancies with twin‐twin transfusion syndrome (TTTS) compared with matched controls with uncomplicated monochorionic diamniotic twin pregnancies, after exclusion of one biological outlier. Red denotes miRNAs with statistically significant fold change difference in TTTS, and blue denotes miRNAs with no significant difference [Colour figure can be viewed at wileyonlinelibrary.com]

A total of 555 of 752 miRNAs were detected; 185 miRNAs were identified in all samples, with a mean of 313 miRNAs detectable per sample. In the PCA, there was a trend towards bimodal clustering of the nine samples (excluding 2022), depending on whether they were TTTS or control samples, which suggests there may be a difference between the groups, although there was still dispersion. The volcano plot demonstrated a number of significantly different miRNA; some were upregulated and some downregulated (Figure 3, significantly differentially expressed shown in red, nonsignificant in blue)**.**

Thirty‐one miRNAs were significantly different in the TTTS compared with control group (see Appendix C for all and Table 3 for “Top 5”). Seventeen miRNAs were upregulated in the TTTS samples, in order of statistical significance: hsa‐miR‐107, hsa‐miR‐519c‐3p, hsa‐let‐7b‐5p, hsa‐miR‐517a‐3p, hsa‐miR‐33a‐3p, hsa‐let‐7c‐5p, hsa‐miR‐519d‐3p, hsa‐miR‐519a‐3p, hsa‐miR‐30b‐5p, hsa‐miR‐518c‐3p, hsa‐miR‐26b‐5p, hsa‐miR‐517c‐3p, hsa‐miR‐99a‐5p, hsa‐miR‐20b‐5p, hsa‐miR‐519b‐3p, hsa‐miR‐144‐3p, and hsa‐miR‐17‐5p; and 14 were downregulated: hsa‐miR‐551a, hsa‐miR‐361‐5p, hsa‐miR‐140‐5p, hsa‐miR‐9‐5p, hsa‐miR‐135a‐5p, hsa‐miR‐128‐3p, hsa‐miR‐450a‐5p, hsa‐miR‐582‐3p, hsa‐miR‐26b‐3p, hsa‐miR‐151a‐5p, hsa‐let‐7d‐3p, hsa‐miR‐556‐3p, hsa‐miR‐223‐3p, and hsa‐miR‐543. They did not remain significant following the Benjamini‐Hochberg correction for multiple testing. A Bayesian model technique was used to examine the differential expression between TTTS and control (Table 4), both unadjusted and adjusted for maternal age, BMI, ethnicity, parity, and gestational age at blood sampling. Neither adjusted nor nonadjusted models demonstrated any significant differential expression between TTTS and control groups by Bayes factor.

**Table 3:** “Top 5” significantly upregulated miRNAs and downregulated miRNAs prior to Benjamini‐Hochberg correction, in pregnancies complicated by twin‐twin transfusion syndrome (TTTS), compared with control of uncomplicated monochorionic diamniotic twin pregnancies

Assay	Average dCq TTTS (SD)	Average dCq Control (SD)	ddCq TTTS—Control	Fold Change TTTS/Control	t Test P Value
hsa‐miR‐107	2.581 (−0.305)	1.765 (0.209)	0.816	1.761	.002
hsa‐miR‐519c‐3p	−3.212 (0.551)	−4.600 (0.373)	1.388	2.618	.003
hsa‐miR‐551a	−2.259 (0.560)	−1.098 (0.219)	−1.160	−2.235	.007
hsa‐let‐7b‐5p	3.219 (0.366)	2.299 (0.409)	0.919	1.891	.012
hsa‐miR‐361‐5p	1.196 (0.175)	1.611 (0.191)	−0.416	−1.334	.014
hsa‐miR‐140‐5p	0.493 (0.145)	1.303 (0.359)	−0.810	−1.753	.015
hsa‐miR‐9‐5p	−6.594 (0.567)	−4.768 (0.529)	−1.826	−3.545	.015
hsa‐miR‐135a‐5p	−6.030 (0.900)	−4.501 (0.331)	−1.529	−2.886	.016
hsa‐miR‐517a‐3p	0.695 (0.522)	−0.228 (0.404)	0.923	1.896	.020
hsa‐miR‐33a‐3p	−4.193 (0.483)	−5.326 (0.608)	1.133	2.193	.024

**Table 4:** Bayesian model of significant miRNAs in pregnancies complicated by twin‐twin transfusion syndrome, compared with uncomplicated monochorionic diamniotic twin pregnancies, unadjusted and adjusted for confounding factors

	logFC	AveExpr	t	P Value	Adj. P Value	B
(a) Unadjusted						
hsa‐miR‐494‐3p	10.78839	7.202803	5.912049	.000116	.01834	−0.02452
hsa‐miR‐204‐5p	9.935406	6.635394	5.731394	.00015	.01834	−0.15169
hsa‐miR‐520c‐3p	8.480225	4.711236	5.070715	.0004	.01834	−0.66378
hsa‐miR‐411‐5p	9.925229	7.538905	4.817742	.000592	.01834	−0.88045
hsa‐miR‐181a‐2‐3p	10.45718	8.027257	4.742297	.000667	.01834	−0.94735
hsa‐miR‐381‐3p	10.75888	8.367614	4.601182	.000834	.01834	−1.07533
hsa‐miR‐518d‐3p	10.82702	8.443192	4.579189	.000864	.01834	−1.09561
hsa‐miR‐369‐5p	10.26154	7.965421	4.576583	.000868	.01834	−1.09802
hsa‐miR‐517‐5p	10.31679	8.030072	4.552294	.000902	.01834	−1.12054
hsa‐miR‐10a‐5p	10.44629	8.173513	4.503198	.000976	.01834	−1.16638
(b) Adjusted^a^						
hsa‐miR‐379‐5p	−186.379	6.396119	−9.89661	.002952	.574946	−4.59509
hsa‐miR‐9‐5p	−197.243	7.054207	−9.77597	.003053	.574946	−4.59509
hsa‐let‐7c‐5p	−81.7966	7.004665	−7.37649	.006547	.574946	−4.59509
hsa‐miR‐577	205.4049	3.385849	6.465976	.009306	.574946	−4.59509
hsa‐miR‐503‐5p	−190.476	6.641287	−5.89464	.011881	.574946	−4.59509
hsa‐miR‐589‐3p	210.6642	7.959102	5.791917	.012443	.574946	−4.59509
hsa‐miR‐365b‐5p	−79.0397	2.389523	−5.55616	.013871	.574946	−4.59509
hsa‐miR‐877‐3p	−81.4977	2.40734	−5.14132	.016964	.574946	−4.59509
hsa‐miR‐219a‐2‐3p	83.26375	1.219888	5.016209	.018075	.574946	−4.59509
hsa‐miR‐548d‐5p	82.48287	1.208447	4.997538	.018249	.574946	−4.59509

a Adjusted for maternal age, BMI, ethnicity, parity, and gestational age at blood sampling.

#### 3.1.5 Identification of candidate miRNAs for validation

Eight miRNAs were identified for validation (Table 5). All miRNAs were detected in all samples in the investigation cohort. No MTI studies were found in maternal serum in pregnancy. See Appendix D for more information on the biological plausibility of the candidate miRNAs.

**Table 5:** Candidate miRNAs for validation

miRNA	Fold Change in Investigation Cohort	Change in TTTS Maternal Serum Compared With Control	Number of Papers With Strong Evidence of Functional MTI^a^	Functional MTI Target Genes^a^	TTTS Biological Plausibility
hsa‐let‐7b‐5p	1.89	Upregulated	42	34	Upregulated in congestive heart failure
hsa‐miR‐17‐5p	1.37	Upregulated	120	80	Associated with angiogenesis
hsa‐miR‐107	1.76	Upregulated	49	38	Upregulated in congestive heart failure, same gene targets as those involved in preeclampsia and spiral artery remodeling
hsa‐miR‐140‐5p	−1.75	Downregulated	37	30	Downregulated in congestive heart failure
hsa‐miR‐223‐3p	−1.59	Downregulated	64	48	Downregulated in stages 3 and 4 chronic kidney disease
hsa‐miR‐517a‐3p	1.90	Upregulated	2	2	Placenta‐specific miRNA
hsa‐miR‐519a‐3p	2.48	Upregulated	12	9	Placenta‐specific miRNA
hsa‐miR‐519c‐3p	2.62	Upregulated	8	6	Placenta‐specific miRNA

Abbreviation: MTI, microRNA‐target interaction.

a According to miRTarBase (Chou C, Shrestha S, Yang C, et al. miRTarBase update 2018: a resource for experimentally validated microRNA‐target interactions. Nucleic Acids Res. 2018;46(D1):D296‐302).

#### 3.1.6 Validation of candidate miRNAs

**Figure 4:**
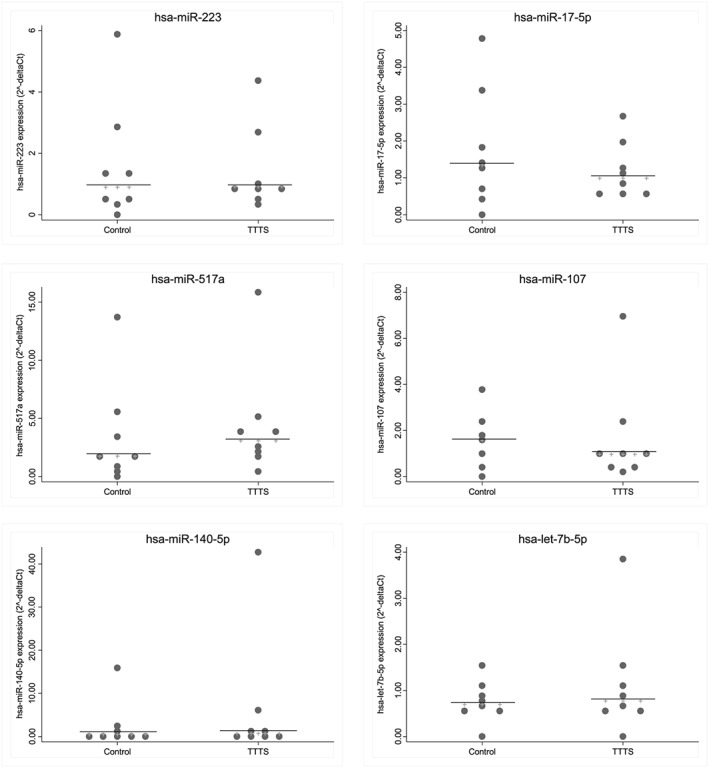
Validation of candidate miRNAs. No significant difference between groups (n = 8 controls, and n = 8 TTTS pregnancies) (Wilcoxon signed‐rank test). Line represents median. miR‐451a used as reference

There was no significant difference in miRNA expression between the TTTS group and the control group (Figure 4). It was not possible to perform RT‐PCR of miR519a‐3p or miR519c‐3p.

## 4 DISCUSSION

This is the first study to compare human maternal serum miRNAs in pregnancies complicated with TTTS and with uncomplicated MCDA twin pregnancies. We found a difference in expression of maternal serum miRNAs in pregnancies complicated by TTTS in the initial investigation cohort. Although the significant difference did not remain following statistical correction for multiple testing, the thresholds for these corrections are naturally conservative, and it was possible our small sample size was associated with a type II statistical error; thus, candidate miRNAs were investigated and validated. None of the candidate miRNAs were confirmed as being significantly different in the validation cohort.

The difference between the investigation cohort and validation cohort may be that the correction for multiple testing was correct, and the initial investigation cohort was too small to demonstrate a statistical difference, particularly as the fold change differences were small. Another reason why the results were not significant is that maternal circulating miRNA may come from different sources (fetus, placenta, or maternal endothelium), and the pathophysiology of TTTS may affect miRNAs from each source differently. The presence of two fetuses with different clinical features may also affect circulating miRNA differently. It may also be that as we were unable to recruit pregnancies with TTTS all at the same stage, that miRNA expression is affected by different stages. In utero intertwin miRNA differences in fetal circulation have not been described, and practically and ethically, it would be difficult to obtain individual fetal blood samples, as an invasive procedure would be required, that would increase the risk of miscarriage in a clinical scenario that is already highly morbid. It may also be that changes in miRNA are a consequence of TTTS, as opposed to a cause of TTTS. Another reason why there may not be a significant difference in miRNA in maternal serum in TTTS pregnancies is that TTTS is a disease that affects the placenta and fetuses, but causes no additional extra‐uterine symptoms, such as maternal proteinuria and hypertension as seen in preeclampsia; therefore, miRNA changes in TTTS may not be seen in maternal circulation. It is interesting to note that while miRNA changes are seen in both maternal plasma and placental tissue in conditions that clinically affect both the mother and the fetus, such as gestational diabetes,^42^, ^43^ and preeclampsia,^27^ in conditions that only affect the fetus, such as fetal growth restriction (FGR), it is not as clear. Studies by Hromadnikova et al have found conflicting changes in miRNA in maternal plasma from FGR pregnancies, with miRNA differences apparent at 12 to 16 weeks' gestation,^31^ but not at 10 to 13 weeks,^44^ thus suggesting miRNA may be gestation dependent. One study looking at miRNA in placental tissue from FGR pregnancies found significant differences,^17^ but another study that compared placental tissue and maternal plasma from the same FGR pregnancies demonstrated changes in placental tissue, but not matched maternal plasma,^16^ despite miRNAs being able to cross into the maternal circulation. It would be interesting to examine miRNA expression in placentas of pregnancies complicated by TTTS; however, there would be major confounders in such a study with many pregnancies having received treatment by FLA that may alter miRNA expression.

A recent study compared miRNA expression in placental samples taken from each twin's umbilical cord root insertion, within monochorionic twin sets in which one twin had FGR and the other twin was normally grown.^13^ This was performed as although monochorionic placentation involves a single placenta, there are differing “vascular territories” between the two fetuses, and this may vary considerably depending upon the severity of the FGR.^45^ Wen et al identified 14 differentially expressed miRNAs (seven upregulated and seven downregulated) in the placental samples of the larger twin compared with smaller twin. These 14 miRNAs were not significantly different between the two normally grown twins in the one monochorionic twin pregnancy, which acted as their control for the miRNA profiling. All 14 miRNAs were successfully validated in a separate unmatched cohort (15 monochorionic twin pregnancies with FGR and 15 monochorionic twin pregnancies with appropriately grown fetuses). Unfortunately, Wen et al did not collect the respective maternal samples to compare with the miRNA expression in the placental samples, but the findings suggest that intertwin differences exist in monochorionic placentas, although this has not been explored in TTTS placentas. It would be interesting to examine miRNAs in maternal blood from sIUGR, particularly those with concurrent TTTS.

It was not possible to perform validation RT‐PCR of the placenta‐specific miRNA, ie, miRNA that are expressed exclusively in the placenta, for reasons which are not clear, although one possible reason is the relative low abundance of placenta‐specific miRNA in maternal serum. As TTTS is a disease that affects the placenta, it may be possible to validate the array findings in these miRNA.

One strength of our study is that the demographics of our patient groups were closely matched, which is vital in miRNA research,^46^ and there was good technical performance in our profiling experiment as demonstrated by the RNA spike‐in and no template control. Unfortunately, the sample size did not allow us to look at the impact of other factors such as pregnancy outcome following TTTS, maternal age, BMI, parity, and fetal sex on miRNA in TTTS, which may have provided further information, although we did produce a Bayesian model to adjust for these factors and attempted to match the samples as closely as possible. Due to the high risk nature of MCDA twins, we were unable to find a group of control MCDA patients with no comorbidities; therefore, we took a pragmatic approach and included two patients with comorbidities (isolated polyhydramnios in one twin at 34 weeks [maximum pool depth, 8.9 cm], pregnancy‐induced hypertension at 34 weeks), which we do not believe will have affected the study results as they are unrelated to TTTS.

## 5 CONCLUSION

This is the first study to look at miRNAs in maternal serum from pregnancies complicated by TTTS. Although the initial profiling array demonstrated a difference in miRNAs in pregnancies complicated by TTTS compared with control MCDA pregnancies, these finding were not confirmed in the validation cohort, although it was not possible to look at the C19MC placenta‐specific miRNA group. Further investigation using alternative biofluids, particularly placental tissue samples, would be interesting, but pragmatically difficult to obtain.

## CONFLICTS OF INTEREST

None declared

## FUNDING SOURCES

F.L.M. is funded by the Richard and Jack Wiseman Trust (charity number 1036690) who had no involvement in the study design, data collection, data analysis, manuscript preparation, and/or publication decision. A.B. is funded by Cancer Research UK.

## DATA AVAILABILITY STATEMENT

The data that support the findings of this study are available from the corresponding author upon reasonable request.

## APPENDIX A ADDITIONAL METHODS

### A.1 RNA extraction

Serum was thawed on ice to room temperature and centrifuged at 3000 *g* for 5 minutes; 200 μL of supernatant from each sample was transferred to new tubes, and 62.5 μL of a well‐mixed master mix of Lysis buffer (60 μL), RNA Spike‐in template mixture (1 μL) (miRCURY LNA Universal RT microRNA PCR, RNA Spike‐in kit [Exiqon]), and carrier‐RNA (1.5 μL) (MS2 RNA, [Roche, West Sussex, UK]) was added. The samples were vortexed and incubated for 3 minutes at room temperature. The Protein Precipitation Solution BF (20 μL) was added to each sample, and the samples were vortexed and incubated for 1 minute at room temperature and then centrifuged at 11 000 *g* for 3 minutes. The clear supernatant was then transferred to a new tube and isopropanol (270 μL) was added, and the sample vortexed to adjust the binding conditions. A miRNA Mini Spin Column BF was placed in a collection tube and the sample loaded onto the column, incubated for 2 minutes at room temperature and centrifuged at 11 000 *g* for 30 seconds. The flow‐through was discarded and the column placed back in the collection tube. To remove residual DNA that may affect downstream applications, Wash Solution 2 BF (700 μL) was added to the spin column BF and centrifuged at 11 000 *g* for 30 seconds. The flow‐through was discarded and the column returned to the collection tube. This was repeated with 250‐μL Wash Solution 2 and was centrifuged at 11 000 *g* for 2 minutes. rDNase (50 μL) was added to the membrane of the spin column and incubated for 15 minutes at room temperature to aid digestion of the DNA. The columns were washed with Wash Solution 1 BF (100 μL), then Wash Solution 2 BF (700 μL), and then Wash Solution 2 BF (250 μL). Following each wash, the samples were centrifuged at 11 000 *g* for 30 seconds, apart from the final wash following that the samples were centrifuged for 2 minutes, to ensure complete drying of the membrane. The spin column was placed in a new collection tube, and RNase free H_2_O (50 μL) was added directly onto the membrane, incubated for 1 minute at room temperature, and centrifuged at 11 000 *g* for 1 minute to elute the samples.

## APPENDIX B miRNAs TESTED IN THIS STUDY

hsa‐miR‐107

hsa‐let‐7b‐5p

hsa‐miR‐30b‐5p

hsa‐miR‐26b‐5p

hsa‐let‐7c‐5p

hsa‐miR‐582‐3p

hsa‐miR‐9‐5p

hsa‐miR‐135a‐5p

hsa‐miR‐519d‐3p

hsa‐miR‐556‐3p

hsa‐miR‐517c‐3p

hsa‐miR‐197‐3p

hsa‐miR‐33a‐3p

hsa‐miR‐361‐5p

hsa‐miR‐517a‐3p

hsa‐miR‐17‐5p

hsa‐miR‐223‐3p

hsa‐miR‐144‐3p

hsa‐let‐7d‐3p

hsa‐miR‐519a‐3p

hsa‐miR‐140‐5p

hsa‐miR‐140‐3p

hsa‐miR‐518c‐3p

hsa‐miR‐501‐3p

hsa‐miR‐519b‐3p

hsa‐miR‐629‐5p

hsa‐miR‐20b‐5p

hsa‐miR‐28‐3p

hsa‐miR‐543

hsa‐miR‐625‐3p

hsa‐miR‐335‐3p

hsa‐miR‐194‐5p

hsa‐miR‐432‐5p

hsa‐miR‐525‐5p

hsa‐miR‐193a‐5p

hsa‐miR‐146a‐5p

hsa‐miR‐26b‐3p

hsa‐miR‐340‐5p

hsa‐miR‐130a‐3p

hsa‐miR‐15a‐3p

hsa‐miR‐1296‐5p

hsa‐miR‐502‐3p

hsa‐miR‐491‐5p

hsa‐miR‐345‐5p

hsa‐miR‐501‐5p

hsa‐miR‐1249

hsa‐miR‐339‐5p

hsa‐let‐7i‐5p

hsa‐miR‐191‐5p

hsa‐miR‐769‐5p

hsa‐miR‐484

hsa‐miR‐1271‐5p

hsa‐miR‐21‐5p

hsa‐miR‐582‐5p

hsa‐miR‐493‐3p

hsa‐miR‐431‐5p

hsa‐miR‐30e‐3p

hsa‐miR‐99a‐5p

hsa‐miR‐181a‐5p

hsa‐miR‐146b‐5p

hsa‐miR‐362‐3p

hsa‐miR‐941

hsa‐miR‐126‐5p

hsa‐miR‐93‐3p

hsa‐miR‐101‐3p

hsa‐miR‐378a‐5p

hsa‐miR‐101‐5p

hsa‐miR‐532‐3p

hsa‐miR‐139‐3p

hsa‐miR‐628‐3p

hsa‐miR‐15b‐5p

hsa‐miR‐376a‐3p

hsa‐miR‐519c‐3p

hsa‐miR‐505‐5p

hsa‐miR‐126‐3p

hsa‐miR‐15b‐3p

hsa‐let‐7f‐2‐3p

hsa‐miR‐33a‐5p

hsa‐miR‐342‐3p

hsa‐miR‐337‐5p

hsa‐miR‐518f‐3p

hsa‐miR‐223‐5p

hsa‐miR‐339‐3p

hsa‐miR‐152‐3p

hsa‐miR‐940

hsa‐miR‐148b‐5p

hsa‐miR‐660‐5p

hsa‐miR‐424‐5p

hsa‐miR‐186‐5p

hsa‐miR‐103a‐3p

hsa‐miR‐125a‐5p

hsa‐miR‐155‐5p

hsa‐miR‐218‐5p

hsa‐miR‐331‐3p

hsa‐miR‐329‐3p

hsa‐let‐7b‐3p

hsa‐miR‐1

hsa‐miR‐335‐5p

hsa‐miR‐127‐3p

hsa‐let‐7g‐3p

hsa‐miR‐509‐3p

hsa‐miR‐518b

hsa‐miR‐551a

hsa‐miR‐515‐3p

hsa‐miR‐598‐3p

hsa‐miR‐196b‐5p

hsa‐miR‐1537‐3p

hsa‐miR‐24‐3p

hsa‐miR‐214‐3p

hsa‐miR‐454‐3p

hsa‐miR‐525‐3p

hsa‐miR‐365a‐3p

hsa‐miR‐148a‐3p

hsa‐miR‐324‐3p

hsa‐miR‐320b

hsa‐miR‐520h

hsa‐miR‐2110

hsa‐miR‐320d

hsa‐miR‐142‐3p

hsa‐miR‐377‐3p

hsa‐miR‐515‐5p

hsa‐miR‐379‐5p

hsa‐miR‐96‐5p

hsa‐miR‐32‐5p

hsa‐miR‐627‐5p

hsa‐miR‐27b‐3p

hsa‐miR‐153‐3p

hsa‐miR‐145‐3p

hsa‐miR‐16‐2‐3p

hsa‐miR‐664a‐3p

hsa‐miR‐542‐5p

hsa‐miR‐618

hsa‐miR‐26a‐5p

hsa‐miR‐550a‐3p

hsa‐miR‐16‐5p

hsa‐miR‐99b‐5p

hsa‐miR‐520a‐5p

hsa‐miR‐450b‐5p

hsa‐miR‐520g‐3p

hsa‐miR‐122‐3p

hsa‐miR‐143‐3p

hsa‐miR‐425‐3p

hsa‐miR‐25‐3p

hsa‐let‐7f‐5p

hsa‐miR‐376a‐5p

hsa‐miR‐30e‐5p

hsa‐miR‐326

hsa‐miR‐376b‐3p

hsa‐miR‐328‐3p

hsa‐miR‐106b‐5p

hsa‐miR‐744‐5p

hsa‐miR‐624‐5p

hsa‐miR‐518c‐5p

hsa‐miR‐370‐3p

hsa‐miR‐524‐5p

hsa‐miR‐106b‐3p

hsa‐let‐7g‐5p

hsa‐miR‐10a‐5p

hsa‐miR‐19b‐1‐5p

hsa‐miR‐106a‐5p

hsa‐miR‐574‐3p

hsa‐miR‐144‐5p

hsa‐miR‐889‐3p

hsa‐miR‐203a

hsa‐miR‐1260a

hsa‐miR‐320a

hsa‐miR‐7‐1‐3p

hsa‐miR‐136‐3p

hsa‐miR‐519e‐3p

hsa‐miR‐30a‐3p

hsa‐miR‐128‐3p

hsa‐miR‐425‐5p

hsa‐miR‐19b‐3p

hsa‐miR‐337‐3p

hsa‐miR‐125b‐5p

hsa‐miR‐221‐5p

hsa‐miR‐132‐3p

hsa‐miR‐502‐5p

hsa‐miR‐654‐5p

hsa‐miR‐188‐3p

hsa‐miR‐30a‐5p

hsa‐miR‐141‐3p

hsa‐miR‐496

hsa‐miR‐20a‐5p

hsa‐miR‐1972

hsa‐miR‐423‐3p

hsa‐miR‐526b‐5p

hsa‐let‐7d‐5p

hsa‐miR‐584‐5p

hsa‐miR‐579‐3p

hsa‐miR‐518e‐5p

hsa‐miR‐301a‐3p

hsa‐miR‐182‐5p

hsa‐miR‐151a‐5p

hsa‐miR‐29a‐5p

hsa‐miR‐224‐5p

hsa‐miR‐143‐5p

hsa‐miR‐23b‐3p

hsa‐miR‐185‐5p

hsa‐miR‐548j‐5p

hsa‐miR‐215‐5p

hsa‐miR‐450a‐5p

hsa‐miR‐22‐3p

hsa‐miR‐100‐5p

hsa‐miR‐29b‐3p

hsa‐miR‐512‐3p

hsa‐miR‐874‐3p

hsa‐miR‐518f‐5p

hsa‐miR‐942‐5p

hsa‐miR‐409‐3p

hsa‐let‐7a‐5p

hsa‐let‐7f‐1‐3p

hsa‐miR‐324‐5p

hsa‐miR‐548k

hsa‐miR‐487b‐3p

hsa‐miR‐16‐1‐3p

hsa‐miR‐154‐5p

hsa‐miR‐524‐3p

hsa‐miR‐195‐5p

hsa‐miR‐654‐3p

hsa‐miR‐205‐5p

hsa‐miR‐24‐2‐5p

hsa‐miR‐199a‐3p

hsa‐miR‐423‐5p

hsa‐miR‐342‐5p

hsa‐miR‐374b‐5p

hsa‐miR‐675‐3p

hsa‐miR‐18b‐5p

hsa‐miR‐21‐3p

hsa‐miR‐505‐3p

hsa‐miR‐30d‐5p

hsa‐miR‐486‐3p

hsa‐miR‐539‐5p

hsa‐miR‐532‐5p

hsa‐miR‐22‐5p

hsa‐miR‐145‐5p

hsa‐miR‐296‐5p

hsa‐miR‐483‐3p

hsa‐miR‐130b‐5p

hsa‐miR‐142‐5p

hsa‐miR‐139‐5p

hsa‐miR‐330‐3p

hsa‐miR‐495‐3p

hsa‐miR‐10b‐5p

hsa‐miR‐518e‐3p

hsa‐miR‐192‐5p

hsa‐miR‐148b‐3p

hsa‐miR‐320c

hsa‐miR‐369‐3p

hsa‐miR‐181b‐5p

hsa‐miR‐545‐3p

hsa‐miR‐34a‐3p

hsa‐miR‐628‐5p

hsa‐miR‐27a‐5p

hsa‐miR‐133b

hsa‐miR‐181c‐3p

hsa‐miR‐375

hsa‐miR‐522‐3p

hsa‐miR‐188‐5p

hsa‐miR‐93‐5p

hsa‐miR‐18a‐3p

hsa‐miR‐766‐3p

hsa‐miR‐210‐3p

hsa‐miR‐376c‐3p

hsa‐let‐7i‐3p

hsa‐miR‐19a‐3p

hsa‐miR‐122‐5p

hsa‐miR‐29c‐3p

hsa‐miR‐652‐3p

hsa‐miR‐382‐5p

hsa‐miR‐33b‐5p

hsa‐miR‐548c‐5p

hsa‐miR‐338‐3p

hsa‐miR‐885‐5p

hsa‐miR‐493‐5p

hsa‐miR‐136‐5p

hsa‐miR‐222‐3p

hsa‐miR‐200b‐3p

hsa‐miR‐193a‐3p

hsa‐miR‐133a‐3p

hsa‐miR‐301b

hsa‐miR‐451a

hsa‐miR‐18a‐5p

hsa‐miR‐95‐3p

hsa‐miR‐424‐3p

hsa‐miR‐23a‐3p

hsa‐miR‐15a‐5p

hsa‐miR‐150‐5p

hsa‐miR‐34a‐5p

hsa‐miR‐27a‐3p

hsa‐miR‐323a‐3p

hsa‐miR‐219a‐5p

hsa‐miR‐193b‐3p

hsa‐miR‐151a‐3p

hsa‐miR‐29c‐5p

hsa‐miR‐181c‐5p

hsa‐miR‐421

hsa‐miR‐29a‐3p

hsa‐miR‐483‐5p

hsa‐miR‐363‐3p

mmu‐miR‐378a‐3p

hsa‐miR‐134‐5p

hsa‐miR‐409‐5p

hsa‐miR‐17‐3p

hsa‐miR‐98‐5p

hsa‐miR‐518a‐3p

hsa‐miR‐190a‐5p

hsa‐miR‐589‐3p

hsa‐miR‐576‐5p

hsa‐miR‐486‐5p

hsa‐miR‐7‐5p

hsa‐miR‐199b‐5p

hsa‐miR‐518d‐5p

hsa‐miR‐183‐5p

hsa‐miR‐551b‐3p

hsa‐miR‐452‐5p

hsa‐miR‐200c‐3p

hsa‐miR‐130b‐3p

hsa‐miR‐877‐5p

hsa‐miR‐485‐3p

hsa‐miR‐30d‐3p

hsa‐miR‐20a‐3p

hsa‐miR‐199a‐5p

hsa‐miR‐548a‐3p

hsa‐miR‐340‐3p

hsa‐miR‐651‐5p

hsa‐miR‐92a‐3p

hsa‐miR‐519e‐5p

hsa‐miR‐374a‐5p

hsa‐miR‐28‐5p

hsa‐miR‐590‐5p

hsa‐miR‐29b‐2‐5p

hsa‐miR‐497‐5p

hsa‐miR‐221‐3p

hsa‐miR‐30c‐5p

hsa‐miR‐410‐3p

hsa‐let‐7e‐5p

hsa‐miR‐516b‐5p

hsa‐miR‐744‐3p

hsa‐miR‐590‐3p

hsa‐miR‐361‐3p

hsa‐let‐7a‐3p

hsa‐let‐7e‐3p

hsa‐miR‐106a‐3p

hsa‐miR‐10a‐3p

hsa‐miR‐10b‐3p

hsa‐miR‐1178‐3p

hsa‐miR‐1181

hsa‐miR‐1183

hsa‐miR‐1185‐5p

hsa‐miR‐1205

hsa‐miR‐1207‐5p

hsa‐miR‐1224‐3p

hsa‐miR‐1227‐3p

hsa‐miR‐1237‐3p

hsa‐miR‐1243

hsa‐miR‐124‐3p

hsa‐miR‐1244

hsa‐miR‐1247‐5p

hsa‐miR‐1253

hsa‐miR‐1254

hsa‐miR‐1255b‐5p

hsa‐miR‐1256

hsa‐miR‐1258

hsa‐miR‐125a‐3p

hsa‐miR‐125b‐2‐3p

hsa‐miR‐1269a

hsa‐miR‐1270

hsa‐miR‐127‐5p

hsa‐miR‐135a‐3p

hsa‐miR‐135b‐5p

hsa‐miR‐137

hsa‐miR‐138‐5p

hsa‐miR‐1468‐5p

hsa‐miR‐146a‐3p

hsa‐miR‐146b‐3p

hsa‐miR‐1471

hsa‐miR‐149‐5p

hsa‐miR‐1538

hsa‐miR‐154‐3p

hsa‐miR‐181a‐2‐3p

hsa‐miR‐181a‐3p

hsa‐miR‐181d‐5p

hsa‐miR‐182‐3p

hsa‐miR‐184

hsa‐miR‐185‐3p

hsa‐miR‐187‐3p

hsa‐miR‐18b‐3p

hsa‐miR‐1908‐5p

hsa‐miR‐190b

hsa‐miR‐1911‐5p

hsa‐miR‐1912

hsa‐miR‐1913

hsa‐miR‐191‐3p

hsa‐miR‐192‐3p

hsa‐miR‐193b‐5p

hsa‐miR‐194‐3p

hsa‐miR‐195‐3p

hsa‐miR‐196a‐5p

hsa‐miR‐196b‐3p

hsa‐miR‐198

hsa‐miR‐19a‐5p

hsa‐miR‐200a‐3p

hsa‐miR‐200b‐5p

hsa‐miR‐204‐5p

hsa‐miR‐206

hsa‐miR‐208b‐3p

hsa‐miR‐20b‐3p

hsa‐miR‐2113

hsa‐miR‐211‐5p

hsa‐miR‐212‐3p

hsa‐miR‐214‐5p

hsa‐miR‐216a‐5p

hsa‐miR‐219a‐2‐3p

hsa‐miR‐224‐3p

hsa‐miR‐23a‐5p

hsa‐miR‐23b‐5p

hsa‐miR‐24‐1‐5p

hsa‐miR‐25‐5p

hsa‐miR‐26a‐1‐3p

hsa‐miR‐26a‐2‐3p

hsa‐miR‐27b‐5p

hsa‐miR‐296‐3p

hsa‐miR‐299‐3p

hsa‐miR‐299‐5p

hsa‐miR‐300

hsa‐miR‐302d‐5p

hsa‐miR‐31‐3p

hsa‐miR‐31‐5p

hsa‐miR‐32‐3p

hsa‐miR‐330‐5p

hsa‐miR‐331‐5p

hsa‐miR‐338‐5p

hsa‐miR‐33b‐3p

hsa‐miR‐346

hsa‐miR‐34b‐5p

hsa‐miR‐34c‐5p

hsa‐miR‐362‐5p

hsa‐miR‐365b‐5p

hsa‐miR‐369‐5p

hsa‐miR‐371a‐3p

hsa‐miR‐373‐3p

hsa‐miR‐373‐5p

hsa‐miR‐374b‐3p

hsa‐miR‐379‐3p

hsa‐miR‐380‐3p

hsa‐miR‐380‐5p

hsa‐miR‐381‐3p

hsa‐miR‐382‐3p

hsa‐miR‐411‐3p

hsa‐miR‐411‐5p

hsa‐miR‐429

hsa‐miR‐431‐3p

hsa‐miR‐433‐3p

hsa‐miR‐449a

hsa‐miR‐449b‐3p

hsa‐miR‐450b‐3p

hsa‐miR‐454‐5p

hsa‐miR‐455‐3p

hsa‐miR‐487a‐3p

hsa‐miR‐491‐3p

hsa‐miR‐494‐3p

hsa‐miR‐498

hsa‐miR‐499a‐5p

hsa‐miR‐500a‐5p

hsa‐miR‐503‐5p

hsa‐miR‐504‐5p

hsa‐miR‐511‐5p

hsa‐miR‐512‐5p

hsa‐miR‐514a‐3p

hsa‐miR‐516a‐3p

hsa‐miR‐516a‐5p

hsa‐miR‐517‐5p

hsa‐miR‐518d‐3p

hsa‐miR‐520a‐3p

hsa‐miR‐520c‐3p

hsa‐miR‐520d‐3p

hsa‐miR‐520d‐5p

hsa‐miR‐520e

hsa‐miR‐521

hsa‐miR‐523‐3p

hsa‐miR‐544a

hsa‐miR‐548a‐5p

hsa‐miR‐548b‐3p

hsa‐miR‐548d‐5p

hsa‐miR‐548e‐3p

hsa‐miR‐548i

hsa‐miR‐548l

hsa‐miR‐548n

hsa‐miR‐550a‐5p

hsa‐miR‐552‐3p

hsa‐miR‐566

hsa‐miR‐567

hsa‐miR‐570‐3p

hsa‐miR‐571

hsa‐miR‐576‐3p

hsa‐miR‐577

hsa‐miR‐589‐5p

hsa‐miR‐592

hsa‐miR‐593‐3p

hsa‐miR‐595

hsa‐miR‐596

hsa‐miR‐601

hsa‐miR‐604

hsa‐miR‐605‐5p

hsa‐miR‐609

hsa‐miR‐610

hsa‐miR‐615‐3p

hsa‐miR‐616‐3p

hsa‐miR‐616‐5p

hsa‐miR‐619‐3p

hsa‐miR‐623

hsa‐miR‐629‐3p

hsa‐miR‐631

hsa‐miR‐635

hsa‐miR‐640

hsa‐miR‐642a‐5p

hsa‐miR‐643

hsa‐miR‐645

hsa‐miR‐646

hsa‐miR‐649

hsa‐miR‐650

hsa‐miR‐655‐3p

hsa‐miR‐661

hsa‐miR‐662

hsa‐miR‐663a

hsa‐miR‐663b

hsa‐miR‐668‐3p

hsa‐miR‐671‐3p

hsa‐miR‐671‐5p

hsa‐miR‐708‐3p

hsa‐miR‐708‐5p

hsa‐miR‐758‐3p

hsa‐miR‐760

hsa‐miR‐761

hsa‐miR‐765

hsa‐miR‐769‐3p

hsa‐miR‐770‐5p

hsa‐miR‐873‐5p

hsa‐miR‐875‐5p

hsa‐miR‐876‐5p

hsa‐miR‐877‐3p

hsa‐miR‐885‐3p

hsa‐miR‐887‐3p

hsa‐miR‐922

hsa‐miR‐92a‐1‐5p

hsa‐miR‐92b‐3p

hsa‐miR‐92b‐5p

hsa‐miR‐933

hsa‐miR‐934

hsa‐miR‐936

hsa‐miR‐937‐3p

hsa‐miR‐9‐3p

hsa‐miR‐99a‐3p

hsa‐miR‐99b‐3p

## APPENDIX C SIGNIFICANTLY DIFFERENT miRNAs, PRIOR TO BENJAMINI‐HOCHBERG CORRECTION, IN PREGNANCIES COMPLICATED BY TWIN‐TWIN TRANSFUSION SYNDROME, COMPARED WITH UNCOMPLICATED MCDA PREGNANCIES

**Table:**

Assay	Average dCq TTTS (SD)	Average dCq Control (SD)	ddCq TTTS—Control	Fold Change TTTS/Control	t Test P Value	Benjamini‐Hochberg FDR
hsa‐miR‐107	2.581 (−0.305)	1.765 (0.209)	0.816	1.761	.002	0.486
hsa‐miR‐519c‐3p	−3.212 (0.551)	−4.600 (0.373)	1.388	2.618	.003	0.486
hsa‐miR‐551a	−2.259 (0.560)	−1.098 (0.219)	−1.160	−2.235	.007	0.494
hsa‐let‐7b‐5p	3.219 (0.366)	2.299 (0.409)	0.919	1.891	.012	0.494
hsa‐miR‐361‐5p	1.196 (0.175)	1.611 (0.191)	−0.416	−1.334	.014	0.494
hsa‐miR‐140‐5p	0.493 (0.145)	1.303 (0.359)	−0.810	−1.753	.015	0.494
hsa‐miR‐9‐5p	−6.594 (0.567)	−4.768 (0.529)	−1.826	−3.545	.015	0.494
hsa‐miR‐135a‐5p	−6.030 (0.900)	−4.501 (0.331)	−1.529	−2.886	.016	0.494
hsa‐miR‐517a‐3p	0.695 (0.522)	−0.228 (0.404)	0.923	1.896	.020	0.494
hsa‐miR‐33a‐3p	−4.193 (0.483)	−5.326 (0.608)	1.133	2.193	.024	0.494
hsa‐let‐7c‐5p	−0.032 (0.516)	−0.856 (0.361)	0.823	1.769	.026	0.494
hsa‐miR‐519d‐3p	−0.868 (0.635)	−2.246 (0.770)	1.377	2.598	.029	0.494
hsa‐miR‐128‐3p	−1.117 (0.250)	−0.460 (0.376)	−0.656	−1.576	.030	0.494
hsa‐miR‐519a‐3p	−2.319 (0.743)	−3.632 (0.697)	1.313	2.484	.031	0.494
hsa‐miR‐30b‐5p	2.585 (0.147)	1.908 (0.383)	0.677	1.599	.032	0.494
hsa‐miR‐518c‐3p	−2.275 (1.176)	−3.958 (0.499)	1.683	3.211	.033	0.494
hsa‐miR‐26b‐5p	0.995 (0.578)	−0.262 (0.736)	1.257	2.390	.033	0.494
hsa‐miR‐517c‐3p	−0.793 (0.602)	−1.828 (0.562)	1.035	2.048	.034	0.494
hsa‐miR‐450a‐5p	−1.175 (0.684)	−0.224 (0.160)	−0.950	−1.932	.034	0.494
hsa‐miR‐582‐3p	−3.722 (0.977)	−2.096 (0.877)	−1.627	−3.088	.035	0.494
hsa‐miR‐99a‐5p	−0.643 (0.421)	−1.375 (0.411)	0.732	1.661	.036	0.494
hsa‐miR‐26b‐3p	−4.091 (0.689)	−2.986 (0.609)	−1.105	−2.151	.039	0.494
hsa‐miR‐151a‐5p	1.935 (0.193)	2.300 (0.219)	−0.365	−1.288	.039	0.494
hsa‐let‐7d‐3p	2.093 (0.480)	2.724 (0.169)	−0.630	−1.548	.040	0.494
hsa‐miR‐20b‐5p	−4.324 (0.561)	−6.309 (1.228)	1.985	3.958	.040	0.494
hsa‐miR‐519b‐3p	−4.841 (0.552)	−6.357 (0.707)	1.516	2.859	.041	0.494
hsa‐miR‐556‐3p	−5.746 (0.547)	−4.483 (0.489)	−1.262	−2.399	.041	0.494
hsa‐miR‐144‐3p	6.066 (0.566)	5.028 (0.637)	1.039	2.054	.042	0.494
hsa‐miR‐223‐3p	9.903 (0.437)	10.570 (0.373)	−0.667	−1.588	.043	0.494
hsa‐miR‐17‐5p	−1.374 (0.353)	−1.832 (0.196)	0.457	1.373	.047	0.515
hsa‐miR‐543	−2.876 (0.797)	−1.899 (0.293)	−0.976	−1.967	.050	0.526

## APPENDIX D BIOLOGICAL PLAUSIBILITY OF THE CANDIDATE miRNAs

The miRNAs in which a difference was revealed in TTTS pregnancies in the investigation cohort may reflect overlapping clinical features often seen in the disease process. Up to 70% of recipient twins have associated cardiac right ventricular dysfunction^47^; interestingly, in adults with congestive heart failure, circulating hsa‐miR‐140‐5p is reported as being downregulated, and hsa‐miR‐107 and hsa‐let‐7b‐5p appears upregulated,^48^ which is what was found in TTTS patients in the investigation cohort. TTTS is also associated with significant changes in renal physiology in both the donor and recipient.^49^, ^50^, ^51^ Circulating hsa‐miR‐223‐3p has been found to be downregulated in adults with chronic kidney disease (stages 4 and 5) (Ulbing, 2017), again echoing the findings in the TTTS investigation cohort.

Approximately 60% of TTTS pregnancies are also affected by fetal growth restriction (FGR),^52^ and changes have been demonstrated in miRNA in placental tissue from FGR singleton pregnancies, although there was no overlap with the miRNA changes demonstrated in the maternal serum samples from the investigation cohort.^53^ However, another study compared placental tissue and maternal plasma from FGR pregnancies and demonstrated changes in placental tissue, but not maternal plasma, despite miRNAs being able to cross into the maternal circulation.^54^ A recent study compared miRNA expression in placental samples taken from each twin's umbilical cord root insertion, within monochorionic twinsets in which one twin had FGR, and the other twin was normally grown.^55^ This was performed as although monochorionic placentation involves a single placenta, there is differing “vascular territories” between the two fetuses, and this may vary considerably depending upon the severity of the FGR.^56^ Wen et al identified 14 differentially expressed miRNAs (seven upregulated and seven downregulated) in the placental samples of the larger twin compared with smaller twin. These 14 miRNAs were not significantly different between the two normally grown twins in the one monochorionic twin pregnancy, which acted as their control for the miRNA profiling. All 14 miRNAs were successfully validated in a separate unmatched cohort (15 monochorionic twin pregnancies with FGR and 15 monochorionic twin pregnancies with appropriately grown fetuses). Unfortunately, Wen et al did not collect the respective maternal blood samples to compare with the miRNA expression in the placental samples, although there was an overlap with four miRNAs that were significantly different in TTTS pregnancies in the investigation cohort: hsa‐let‐7c, hsa‐miR‐518c‐3p, hsa‐miR‐144‐3p, and hsa‐miR‐543. hsa‐let‐7c was upregulated in TTTS pregnancies in the investigation cohort and upregulated in the larger twin in Wen study; however, the changes in the other three miRNAs were in line with the smaller twin, not the larger twin: hsa‐miR‐518c‐3p and hsa‐miR‐144‐3p were upregulated in TTTS and upregulated in the smaller twin, and hsa‐miR‐543 was downregulated in TTTS and downregulated in the smaller twin, indicating that an association may exist between miRNA expression in placental samples and maternal serum and TTTS and FGR.

Changes were also demonstrated in the investigation cohort in hsa‐miR‐517a‐3p, hsa‐miR‐519a‐3p, and hsa‐miR‐519c‐3p, which are members of the C19MC group of placenta‐specific miRNAs,^57^, ^58^ and are detectable from the first trimester in chorionic villi stromal cells.^59^ hsa‐miR‐17‐5p is known to be associated with angiogenesis^60^ and increased hsa‐miR‐17 and hsa‐miR‐20b expression with reduced spiral artery remodeling^61^ and preeclampsia.^62^ A recent study has found that downregulation of hsa‐miR‐17‐5p improves cardiac function after myocardial infarction, thought to be due to aiding repair of vascular injury, and decreasing the rate of apoptosis.^63^ Other miRNAs that were significant prior to correction for multiple testing have also been demonstrated to be significantly different in maternal serum from singleton pregnancies complicated by preeclampsia: hsa‐miR‐107, hsa‐miR‐223, hsa‐miR‐26b, hsa‐miR‐30b, hsa‐miR‐517c, hsa‐miR518c, hsa‐miR‐519d, and hsa‐miR‐99a.^64^, ^65^ This again fits with issues of placentation and abnormal placental vasculogenesis seen in TTTS, although the majority of this work has been performed in singleton pregnancies.

With regard to potential gene targets of the differentially expressed miRNA in the investigation cohort identified from miRTarBase, although there is a paucity of gene target validation studies in pregnancy, the potential gene targets do have biological plausibility, for example, miR‐107 targets include NOTCH2 and DICER1, which have been shown to be involved in preeclampsia and spiral artery remodeling,^66^, ^67^ which supports further investigation of these miRNAs in pregnancy to both to improve knowledge of the pathophysiology of TTTS and also to enable further study of miRNAs as potential biomarkers of TTTS.
